# Citrate Synthase Knockdown Suppresses Cell Proliferation and Induces Apoptosis in Select Human Cancer Cell Lines

**DOI:** 10.3390/ijms27010083

**Published:** 2025-12-21

**Authors:** Xiaoxiao Zhang, Siyu Qu, Huanhuan Zhong, Yulu Yang, Bo Cheng, Yan Zeng

**Affiliations:** Department of Zoology, College of Life Sciences, Nanjing Agricultural University, Nanjing 210095, China

**Keywords:** citrate synthase, the TCA cycle, the cell cycle, apoptosis

## Abstract

Citrate synthase (CS) catalyzes the first reaction in the tricarboxylic acid (TCA) cycle and is one of the rate-limiting and regulatory enzymes of the TCA cycle. How CS influences human cells beyond its direct roles in carbohydrate metabolism and energy production is poorly understood. In this study, we used RNA interference (RNAi) to knockdown CS expression in three diverse human cancer cell lines, HCT116, HT-1080, and HepG2, and assessed changes in their cellular behaviors. In all three cell lines, the loss of CS led to are duction in cell proliferation, increased apoptosis, lower mitochondrial membrane potentials, higher reactive oxygen species (ROS) production, and reduced ATP levels. We then performed transcriptome analyses in the three cell lines, identified pathways related to the cell cycle and apoptosis that might elucidate the mechanisms underlying those cellular changes, and further verified the mRNA expression changes in specific genes associated with the apoptotic pathways. Taken together, our results suggest that CS regulates a broad spectrum of human cellular processes.

## 1. Introduction

CS (E.C.2.3.3.1) is an enzyme located in the mitochondrial matrix that catalyzes the formation of citrate from oxaloacetic acid and acetyl coenzyme A [1]. This is the crucial entry point or the first step in the TCA cycle, which, when coupled with the electron transfer chain and oxidative phosphorylation, generates the majority of ATP under aerobic conditions. The TCA cycle also plays a key role in both catabolic and anabolic processes by providing intermediates for the synthesis of sugars, lipids, and amino acids [2]. Biochemically, CS is one of the rate-limiting enzymes of the TCA cycle, but whether and to what extent CS influences cellular processes beyond its functions in carbon and energy metabolism has not been well characterized. In certain species of mice, a CS allele that resulted in a partial loss of CS activity might contribute to age-related hearing loss, even though the exact mechanism is unknown [3,4,5,6]. Given that metabolism is intimately associated with tumorigenesis, previous studies had also examined CS’s roles in cancers. Increased CS activity and expression have been reported in human pancreatic cancer, lymphoma, and ovarian cancer [7,8,9]. But when the RNAi technique was used to investigate the effects of CS knockdown on the behaviors of human cells, including cancer cell lines, conflicting results were obtained. In the human cervical carcinoma cell line HeLa, RNAi against CS enhanced cell proliferation and metastasis [10]. Yet in ovarian cancer cell lines SKOV3 and A2780, CS RNAi reduced cell proliferation, inhibited cell migration, and modestly increased ATP production [9]. In the human glioblastoma cell line SF188, the suppression of CS prevented apoptosis [11]. On the other hand, CS downregulation in the human embryonic kidney cell line 293T resulted in lower ATP levels and higher superoxide formation and apoptosis [12]. These controversial results indicated that CS indeed impacted various aspects of cell physiology, but our understanding of its roles and mechanisms remains rudimentary, and a more systematic analysis is required to resolve the discrepancies.

So, in this study, we used three diverse human cancer cell lines, HCT116, HT-1080, and HepG2, as examples to study the effects of RNAi-mediated CS reduction on cellular behaviors such as proliferation and apoptosis. High-throughput RNA sequencing (RNA-seq) experiments and data analyses were then performed to identify gene expression and pathway changes that might shed light on the mechanisms underlying the observed cellular phenotypes.

## 2. Results

### 2.1. RNAi-Mediated Knockdown of CS Expression

Our initial interest in CS stemmed from the fact that CS was predicted by multiple microRNA target programs to be a miR-122 target, which was confirmed using a reporter assay [13]. To further characterize this relationship, the publicly available gene expression datasets from miR-122 knockout mouse studies were examined [14,15]. In both the GSE20610 [14] and GSE27713 [15] datasets, CS mRNA levels were higher in the livers of knockout mice than in the wildtype controls (Figure S1A,B), consistent with the expectation that miR-122 inhibits CS expression in vivo. Conversely, when miR-122 was introduced into 293T or HepG2 cells, CS mRNA was reduced (GSE123311, Figure S1C, [13]; Figure S2). Finally, we identified the miR-122 binding site in CS mRNA, constructed a luciferase reporter plasmid (Figure S3, left panel), and further showed that deletion of the binding site sequence abrogated miR-122 inhibition of CS expression in the reporter assay (Figure S3, right panel). While this work was ongoing, a paper was published showing that miR-122 regulates CS in nasopharyngeal carcinoma [16].

**Figure 1:**
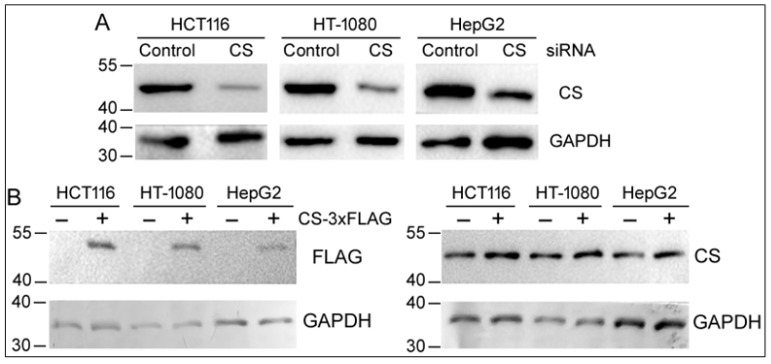
Knocking down and overexpressing CS in human cell lines. (**A**) RNAi-mediated knockdown of CS expression in HCT116, HT-1080, and HepG2 cells. Cells were transfected with a control siRNA or CS siRNA for 4 days (HT-1080 and HepG2) or 6 days (HCT116). Proteins were extracted and Western blotting was performed to detect CS or GAPDH (as a loading control). Positions of the protein markers in kilodaltons are indicated on the left. (**B**) CS overexpression in HCT116, HT-1080, and HepG2 cells. Cells were transfected with the pCMV-CS-3xFLAG plasmid for 2 days before Western blotting using antibodies against FLAG, CS, or GAPDH. Positions of the protein markers in kilodaltons are shown on the left. The left and right panels represent two separate blots with the same protein samples loaded on a gel.

miR-122 is predominantly expressed in the liver, but the TCA cycle and CS are essential in most cell and tissue types. As the effects of CS knockdown have been controversial in SKOV3, A2780, HeLa, SF188, and 293T cells [9,10,11,12], we decided to look more closely at the roles of CS in three popular and diverse human cancer cell lines: HCT116, HT-1080, and HepG2. HCT-116 is a colon cancer cell line, HT-1080 is a fibrosarcoma cell line, and HepG2 is a hepatocellular carcinoma. All three cell lines have been extensively used in biological and pharmacological studies. We first used a small interfering RNA (siRNA) to transiently knockdown CS expression. Figure 1A shows that CS protein expression was reduced by approximately 80% in all three cell lines 4–6 days after siRNA transfection. Conversely, we also tried to overexpress CS by transfecting cells with a 3xFLAG-tagged CS plasmid. Since CS is synthesized in the cytosol and imported to the mitochondrial matrix, its N-terminus contains the important mitochondrial target sequence [1,17]. We therefore appended the 3xFLAG epitope to the C-terminus of CS. Two days after the plasmid transfection, Western blotting was performed, detecting specific CS-3xFLAG expression using the FLAG antibody (Figure 1B). But when a CS-specific antibody was used, the vast majority of the signals came from the endogenous CS protein, judged from the migration patterns of the endogenous and recombinant CS proteins (Figure 1B). Despite repeated attempts, including using other cell lines such as 293T cells, we were never able to express the CS-3xFLAG protein at a level close to that of the endogenous CS.As a result, subsequent studies focused on RNAi against CS and examined the cells six days after siRNA transfection, unless indicated otherwise.

### 2.2. Characterizing the Effects of CS Knockdown on the Cellular Phenotypes

**Figure 2:**
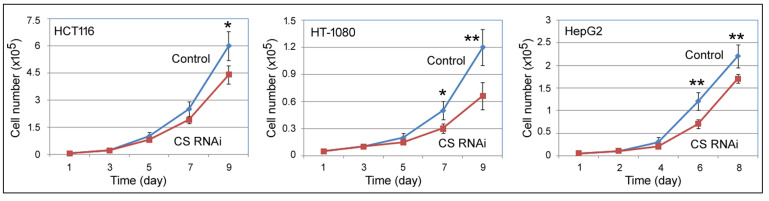
Growth curves of cells undergoing a control or CS RNAi. The indicated cell lines were transfected with a control or CS siRNA, and cell growth was measured by MTT assays. The *y*-axis shows the cell number, and the *x*-axis shows the time in days. Symbols represent average cell numbers, with standard deviations indicated on the graphs. Comparing the control and CS RNAi, *: *p* < 0.05; **: *p* < 0.01.

**Figure 3:**
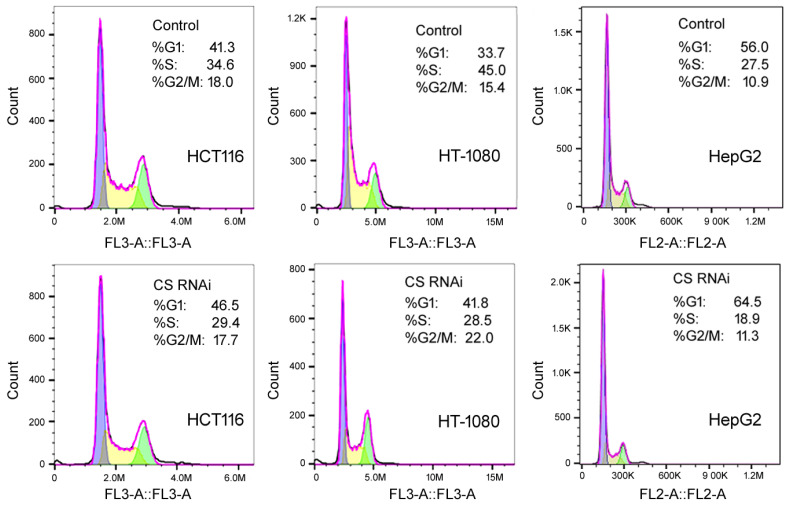
The cell cycle profiles of cells treated with a control siRNA (top panels) or CS siRNA (bottom panels) for 6 days. The *y*-axis is the cell count, the *x*-axis is PI (propidium iodide) staining for DNA content in fluorescence units as determined by flow cytometry. Representatives of at least three experiments are shown here. The percentages of cells in the G1, S, and G2/M phases were determined automatically by the default settings of the flow cytometer and software and listed inside the graphs.

**Figure 4:**
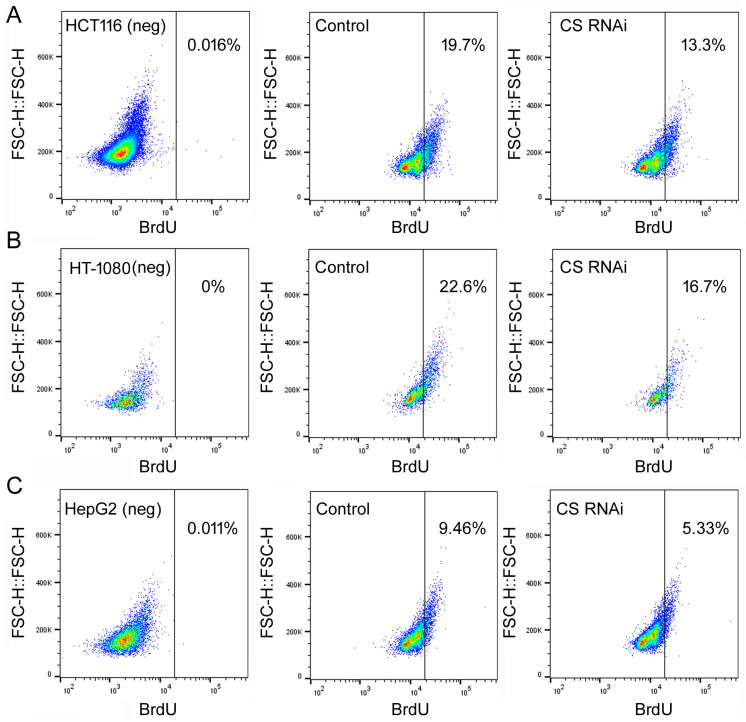
BrdU labeling of cells undergoing a control or CS RNAi. HCT116 (**A**), HT-1080 (**B**), and HepG2 (**C**) cells were transfected with a control or CS siRNA for 6 days, then treated with 10 M BrdU for 60 min before being processed for FITC-BrdU antibody staining and cell flow cytometry. The *y*-axis is cell scattering; the *x*-axis is FITC signals for BrdU incorporation in units determined by flow cytometry. Representatives of three experiments are shown here. The left panels are samples of cells not treated with BrdU, thus serving as the negative staining controls and setting up a gate for BrdU-positive cells on the right. The middle panels are cells transfected with the control siRNA and treated with BrdU. The right panels are cells transfected with the CS siRNA and treated with BrdU. Percentages of BrdU-positive cells are indicated in the graphs.

To examine how the loss of CS influenced cell behaviors, MTT (thiazolyl blue tetrazolium bromide) assays were first used to monitor cell number increase in cells transfected with the control or CS siRNA on day 0, for up to 9 days, as described previously [18]. In all three cell lines, HCT116, HT-1080, and HepG2, CS RNAi led to slower cell growth (Figure 2). Their cell cycle profiles on day 6 were then examined by flow cytometry. Day 6 was chosen since this was the time with the most CS suppression (Figure 1A). Representative results are shown in Figure 3. Compared to the control siRNA transfections (upper panels), the most conspicuous change in all three cell lines was the reduction inS-phase cells by CS RNAi (Figure 3, lower panels). To further examine the effect of CS knockdown on S phase transition, we performed pulse BrdU (5′-bromo-2′-deoxyuridine) labeling experiments. Cytometry analyses indicated that cells transfected with the CS siRNA had fewer BrdU-labeled cells than those transfected with the control siRNA (Figure 4A–C, compare the right panels to the middle panels). For example, the percentage of the gated population of BrdU-positive cells in the control HCT116 sample was 19.7%, while in the CS RNAi sample was 13.3% (Figure 4A), corresponding well with their cell cycle profiles (Figure 3, left panels). These cell cycle results matched the MTT data, suggesting that CS knockdown reduced cell proliferation and cell cycle progression, including the hindrance of S phase transition.

**Figure 5:**
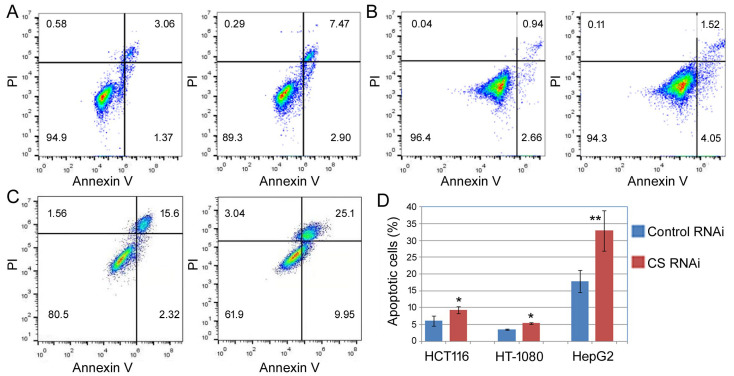
Apoptosis analyses of cells after CS knockdown. HCT116 (**A**), HT-1080 (**B**), and HepG2 (**C**) cells were transfected with a control (left panels) or CS siRNA (right panels) for 6 days, then subjected to PI and Annexin V staining and flow cytometry analyses. PI (the *y*-axis) detected DNA content, and Annexin V (the *x*-axis) examined apoptosis in fluorescence units as determined by flow cytometry. Apoptosis was represented by the lower right quadrant and the upper right quadrant, corresponding to the early and late apoptotic cells, respectively. Percentages of cells in each quadrant were determined automatically by the flow cytometry software and are presented in the graphs. (**D**) Averages and standard deviations of the percentages of total apoptotic cells. Comparing the control and CS RNAi, *: *p* < 0.05; **: *p* < 0.01.

Next, we examined apoptosis using Annexin V staining and flow cytometry. High Annexin V staining signifies apoptotic cells. As shown in Figure 5, even though the three cell lines differed in the extent of apoptosis, e.g., HepG2 (Figure 5C) had more apoptotic cells than HCT116 (Figure 5A) and HT-1080 (Figure 5B), CS knockdown invariably increased the ratios of apoptotic cells (Figure 5A–D).

**Figure 6:**
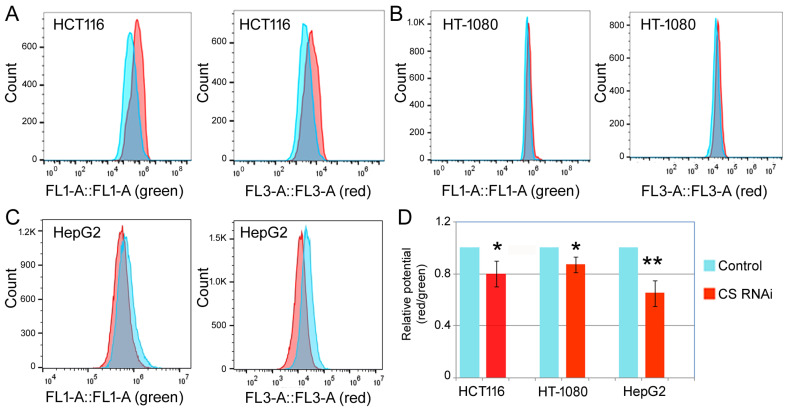
Relative mitochondrial membrane potentials of the cells undergoing the control and CS RNAi. HCT116 (**A**), HT-1080 (**B**), and HepG2 (**C**) cells were transfected with a control or CS siRNA for 6 days, then the mitochondrial membrane potentials were examined by flow cytometry analyses. The *y*-axis is cell counts; the *x*-axis is green or red fluorescence signals in fluorescence units as determined by the flow cytometer. The left panels are green fluorescence signals from JC-1 monomers, and the right panels are red fluorescence signals from JC-1 aggregates. Blue peaks are signals of the control cells; red peaks are CS RNAi cells. (**D**) Averages and standard deviations of the relative mitochondrial membrane potentials, represented by the ratios of the red vs. green fluorescence signals. Control cells were set at 1. Comparing the control and CS RNAi, *: *p* < 0.05; **: *p* < 0.01.

**Figure 7:**
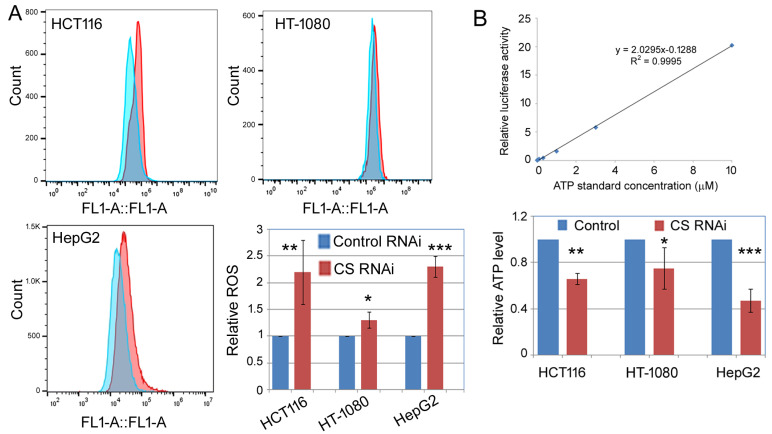
ROS and ATP levels in CS knockdown cells. (**A**) The indicated cells were transfected with a control or CS siRNA for 6 days, then processed and stained with 2,7-Dichlorodi-hydrofluorescein diacetate, which reacts with ROS and emits green fluorescence. The *y*-axis is cell counts; the *x*-axis is green fluorescence signals in fluorescence units determined by flow cytometry. The bar graph summarizes the quantification of relative ROS level (the *y*-axis) with that of the control RNAi cells set at 1. Averages and standard deviations of at least three biological replicates are shown. (**B**) Quantification of ATP levels in the control and CS RNAi cells. The top panel shows an ATP standard curve assayed using a luminometer, showing the dynamic range within which our sample readings fell. The bottom bar graph presents the relative ATP levels (the *y*-axis), with that of the control RNAi cells set at 1. Averages and standard deviations of at least three biological replicates are shown. Comparing the control and CS RNAi cells, *: *p* < 0.05; **: *p* < 0.01; ***: *p* < 0.001.

The biochemical activity of CS lies in the metabolism and energy production in the mitochondria, and many cellular phenotypes, such as apoptosis, are also closely linked to mitochondrial functions [19]. Thus, we measured the relative mitochondrial membrane potential, and the intracellular ROS and ATP levels upon RNAi against CS. Figure 6 presents the results of relative mitochondrial membrane potential studies. The left panels in Figure 6A–C show the green fluorescence by the JC-1 dye monomers, formed under the low membrane potential conditions. The right panels in Figure 6A–C show the red fluorescence by the JC-1 aggregates, formed under the high membrane potential conditions. The ratio between the red and green fluorescence then serves as an index of relative changes in the mitochondrial membrane potentials (Figure 6D). Our data showed that CS RNAi led to a modest but significant reduction in relative mitochondrial membrane potential in HCT116, HT-1080, and HepG2 cells (Figure 6). On the other hand, ROS levels (Figure 7A) were consistently elevated, and ATP levels (Figure 7B) were suppressed by CS RNAi.

### 2.3. The Effects of CS Knockdown on Gene Expression in Cancer Cells

**Figure 8:**
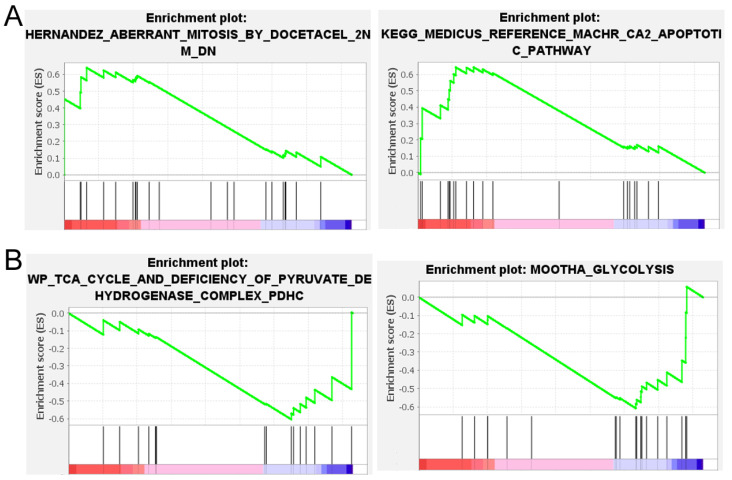
Gene sets enriched in the low CS (**A**) and high CS (**B**) expressing cells. GSEA compared RNA-seq data from cells with the control and CS RNAi, and gene sets related to the cell cycle, apoptosis, and glucose metabolism, which are among the top 10 enriched in both categories, are shown.

As shown above, CS knockdown induced a wide range of phenotypic changes in human cells. To appreciate the underlying mechanisms, RNA-seq was carried out on HCT116, HT-1080, and HepG2 cells undergoing CS RNAi on day 6. Our goal was to identify mRNA expression changes applicable to as many different kinds of cells as possible. To this end, GSEA was then performed comparing the control and CS RNAi conditions [20]. In cells with low CS expression, gene sets corresponding to aberrant mitosis and apoptotic pathways were enriched (Figure 8A), consistent with the cell cycle and apoptosis phenotypes we observed (Figure 3, Figure 4 and Figure 5). In cells with high CS expression, i.e., control RNAi, gene sets associated with the TCA cycle and glycolysis (Figure 8B) were enriched, likely reflecting normal metabolism in these control cells.

We also derived differentially expressed genes common in all three cell lines (Table S1) and then subjected them to functional, gene ontology (GO), and pathway analyses [21]. Table 1 shows the top 10 terms in the UP_KW_BIOLOGICAL_PROCESS category. Two of them, KW-0498 (Mitosis) and KW-0159 (Chromosome partition), concurred with the cell cycle changes upon CS knockdown. A number of processes linked to lipid metabolism were also present, which might be explained by the fact that the TCA cycle provides intermediates for the synthesis of lipids. Table 2 shows the top 10 terms in the GOTERM_BP_DIRECT category. Here, GO:0007059 (chromosome segregation) and GO:0043066 (negative regulation of apoptotic process) were prominently featured, and GO:0042149 (cellular response to glucose starvation) might reflect the metabolic perturbation resulting from CS suppression. Table 3 shows the top 10 terms in the KEGG_PATHWAY category. The hsa04137:Mitophagyterm is at the top of the list, likely revealing responses to impaired mitochondria due to the CS knockdown and TCA cycle disruption. Most of the rest were metabolism-related pathways, e.g., hsa01100 (Metabolic pathways) and hsa01200 (Carbon metabolism). Table 3 also lists the BIOCARTA category, with all three terms being apoptosis-associated pathways. Taken together, these analyses confirmed the loss of CS and the resulting metabolic disturbance, while providing plausible mechanistic explanations for the cell cycle and apoptosis phenotypes.

### 2.4. Validating Specific mRNA Expression

**Figure 9:**
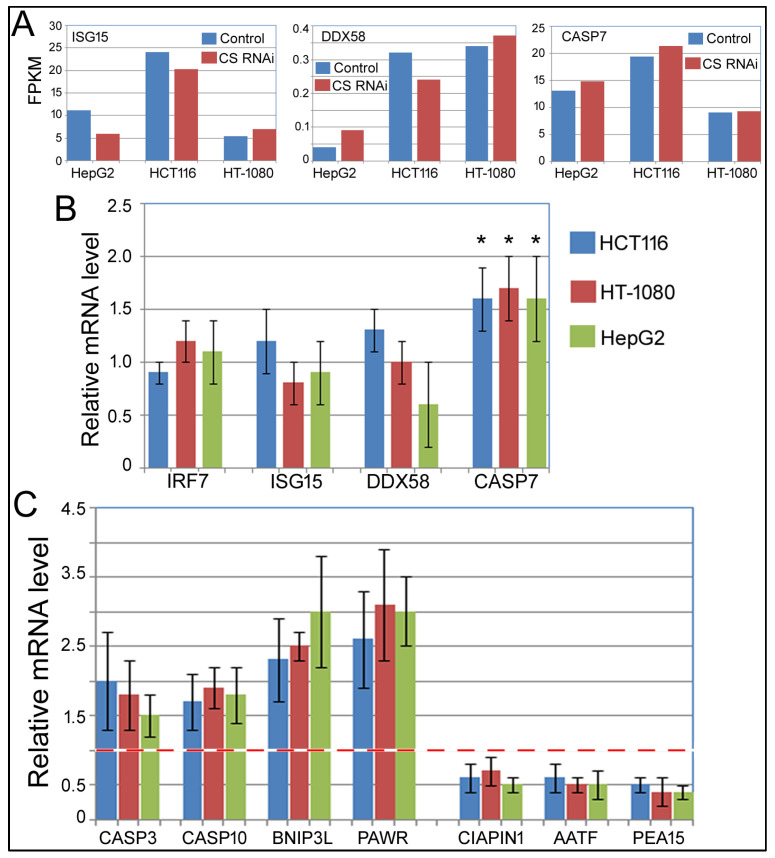
Individual gene expression. (**A**) Expression of ISG15, DDX58, and CASP7, according to GSE306823. The *Y*-axis is the FPKM values by RNA-seq. (**B**) qPCR analyses of the mRNA expression of IRF7, ISG15, DDX58, and CASP7 in HCT116, HT-1080, and HepG2 cells transfected with a control or CS siRNA for 6 days. The *Y*-axis is the relative mRNA expression, with that in the control siRNA transfection condition set at 1; thus, only the relative expression of the CS siRNA transfection is shown in the graph. Averages and standard deviations of three biological replicates are shown. Comparing the CS RNAi conditions with 1, the control RNAi conditions, *: *p* < 0.05. (**C**) qPCR analyses of the mRNA expression of the indicated genes in HCT116, HT-1080, and HepG2 cells transfected with a control or CS siRNA for 6 days. Plot descriptions are the same as in (**B**). The red line marks 1.0 on the *y*-axis. Comparing the relative mRNA levels in CS RNAi samples with the control RNAi samples (set at 1), all these genes had *p* < 0.05.

In ovarian cancer cell lines SKOV3 and A2780, CS knockdown increased the mRNA levels of ISG15, IRF7, CASP7, and DDX58, according to microarray and qPCR studies [9]. These genes were not identified as differentially expressed in our RNA-seq dataset (GSE306823, Table S1). By RNA-seq, the IRF7 mRNA level was too low to be reliably detected in HepG2 cells, and Figure 9A shows that ISG15 and DDX58 were not increased by CS RNAi, although CASP7 did show a trend of increase in all three cell lines. To test their expression in depth, we performed qPCR analyses in HCT116, HT-1080, and HepG2 cells. Figure 9B showed that IRF7, ISG15, and DDX58 mRNA levels were still not increased by CS RNAi, but CASP7 was elevated by an average of approximately 60% in all three cell lines. Since CASP7 (caspase 7) is involved in apoptosis, and CS knockdown enhanced apoptosis (Figure 5 and Figure 8, Table 2 and Table 3), we decided to investigate the mRNAs of a number of apoptosis-related genes in our cells in response to RNAi against CS. Not all apoptotic genes altered their mRNA expression, but a significant number of them did, and some were already identified as differentially expressed by RNA-seq (Table S1). A few examples are shown in Figure 9C. CASP3 (caspase 3) and CASP10 (caspase 10), functioning similarly to CASP7, likewise increased their expression. Other pro-apoptotic genes, such as BNIP3L (BCL2 interacting protein 3 like [22]) and PAWR (pro-apoptotic WT1 regulator [23]), were also stimulated in CS knockdown cells (Table S1; Figure 9C). On the other hand, anti-apoptotic genes, such as CIAPIN1 (cytokine-induced apoptosis inhibitor 1, [24]), AATF (apoptosis-antagonizing transcription factor [25]), and PEA15 (proliferation and apoptosis adaptor protein 15 [26]), had their mRNA expression reduced in CS knockdown cells (Table S1 and Figure 9C). These qPCR studies thus verified our RNA-seq results and suggested that changes in these genes’ expression contribute to the increased apoptosis phenotype upon CS knockdown in the cancer cells.

## 3. Discussion

This study investigated the cellular and gene expression changes in three human cancer cell lines, HCT116, HT-1080, and HepG2, upon RNAi-mediated knockdown of CS expression. Our results showed that CS knockdown reduced cell proliferation, cell cycle progression, mitochondrial membrane potential, and ATP levels but enhanced apoptosis and cellular ROS.RNA-seq and qPCR analyses then identified changes in biological processes, pathways, and specific genes that might provide the mechanisms underlying the cell cycle and apoptosis phenotypes.

All previous studies examined the effects of CS knockdown. Our work also attempted to overexpress a recombinant CS-3xFLAG protein. While the protein was detected using a FLAG antibody, the overall level of CS protein barely changed (Figure 1B). Due to the housekeeping functions of CS and the TCA cycle, it is possible that CS is already expressed so proficiently that it is difficult to generate more CS protein. Alternatively, enzymes in the TCA cycle might work in a narrow, stoichiometric range concertedly, and the process of mitochondrial import adds an extra checkpoint on how much the enzymes can be maintained. Thus, besides the well-known allosteric regulation, CS might also be controlled at the gene expression level. As CS was not overexpressed appreciably, we relied on RNAi to investigate the contribution of CS to cellular behaviors.

Previous studies of CS RNAi had yielded conflicting results. It was reported to enhance cell proliferation and metastasis in HeLa cells [10], yet the opposite effects were seen in SKOV3 and A2780 cells [9]. Apoptosis was suppressed in SF188 cells [11] but elevated in 293T cells [12]. ATP production was higher in one study [9] but lower in others [10,12]. Thus, how CS loss influences biological processes merits more systematic analyses. Unlike previous studies that relied on a single cell line or cell lines from the same cancers, this work utilized cell lines derived from three different human cancers. Consistent results were obtained in these three cell lines on cell proliferation, apoptosis, mitochondrial membrane potential, ROS, and ATP levels (Figure 2, Figure 3, Figure 4, Figure 5, Figure 6 and Figure 7). Our results matched more closely with those in the 293T cells in terms of increasing apoptosis and decreasing ATP levels [12]. They also agreed with the study on SKOV3 and A2780 cells [9] regarding the inhibitory effect on cell growth, but the pro-apoptotic effects were more pronounced in our work, and we observed lower ATP instead of higher ATP with CS knockdown. Interestingly, it had been reported that citric acid induced growth inhibition, inflammation, and apoptosis in cultured human hair follicle and dermal papilla cells, and that CS knockdown reversed the phenotypes, albeit whether CS knockdown alone elicited any physiological changes was not examined in detail [27]. Discrepancies in published reports may stem from the varying times of siRNA treatments, the different cell lines and experimental systems employed, and the different assays performed. But considering that we examined diverse cell lines with results largely overlapping with those from the SKOV3 and A2780 ovarian cancer cells and 293T cells, a non-cancer cell line, our conclusions should be more widely applicable to human cells. Our observed effects on cell proliferation and apoptosis are also consistent with the hearing loss phenotype in mice with a hypomorphic CS allele [4,5,6], although other possibilities cannot be ruled out.

Results from this study and others [9,10,11,12,27] thus demonstrated that CS, a crucial enzyme in the TCA cycle, through direct or indirect means, impinges on other cellular processes as well. Much interest in CS originates from the important connection between tumorigenesis and metabolism [2,28], the fact that CS is a critical regulatory factor in the TCA cycle [1], and the reported CS changes in human cancers [7,8,9]. Analyses using the more recent and comprehensive data from the Cancer Genome Atlas Program (https://www.cancer.gov/tcga, accessed on 7 November 2025) revealed that CS expression is altered in certain cancers, including CHOL (cholangiocarcinoma) and LIHC (liver hepatocellular carcinoma) (Figure S4, [29]). Since CS knockdown inhibited cell proliferation and stimulated apoptosis, this strategy might be explored to treat such cancers in the future. Considering the essential role of CS in metabolism, interfering with CS function should be used in a targeted manner or to supplement other therapeutics.

Another new feature of our work is that we performed a much more detailed transcriptomics analysis to gain mechanistic insights into the observed phenotypes. Figure 8 shows that cells with low CS had gene sets enriched in aberrant mitosis and apoptotic pathways, and cells with high CS, i.e., the “normal” cells, enriched gene sets in the TCA cycle and glycolysis. Functional, GO, and pathway analyses also verified differences in the cell cycle, apoptosis, and metabolism between the control siRNA-treated cells and the CS siRNA-treated cells (Table 1, Table 2 and Table 3). These results, from a different angle, confirmed the loss of CS and its effects on metabolism and provided plausible explanations for the decreased cell growth and increased apoptosis in cells with CS knockdown.

Much more work is needed to understand how CS affects cell proliferation, apoptosis, mitochondrial membrane potential, ROS, and ATP production. The loss of CS, and presumably the reduction in TCA cycle activity, can have a direct and indirect, pleiotropic effect on cells due to changes in the levels of ATP and carbohydrates that are crucial in sugar, lipid, and amino acid synthesis. Some of these phenotypes might also be interconnected. One can imagine that lower CS puts the brakes on the TCA cycle, causing an imbalance in carbon flux of sugar and impeding the production of ATP in the mitochondria. Disrupted metabolism in the mitochondria could depress the mitochondrial membrane potential but induce ROS. Changes in the mitochondria might initiate the apoptotic pathway [19,30]. Since DNA replication and mitosis are energy-intensive processes, ATP deficiency would hinder the cell cycle progression (Figure 2, Figure 3 and Figure 4), with apoptosis likely further reducing cell proliferation (Figure 2). Interestingly, GSEA, GO, and pathway analyses prominently yielded results linked to apoptosis (Figure 8A, Table 2 and Table 3), and many such related genes were differentially expressed upon CS RNAi (Table S1). By qPCR, we validated their mRNA changes. In particular, the mRNAs of pro-apoptotic genes, such as CASP3, CASP7, CASP10, BNIP3L, and PAWR, were increased in CS knockdown cells, and, conversely, those of the anti-apoptotic genes, such as CIAPIN1, AATF, and PEA15, were decreased (Table S1 and Figure 9). While future work should dissect whether or how any of these genes participate in apoptosis, such results at least support the finding that insufficient CS induced apoptosis and shed light on the potential mechanisms. Notably, many of these genes, such as CIAPIN1, AATF, and PEA15, likewise play a role in cell proliferation and cell cycle progression [31,32,33].

CS knockdown could also impact other aspects of cell physiology. For example, KEGG analysis identified differential gene expression changes in the mitophagy pathway (Table 3), suggesting that mitophagy or autophagy in general might be affected as well [34]. Another cell death pathway is necrosis [34,35]. The mRNA expression of prominent necrosis regulators, such as RIPK1, RIPK3, MLKL, GPX4, and FSP1, was not altered by CS RNAi (GSE306823, Table S1), and bioinformatics analyses did not reveal enrichment or changes in necrosis or inflammation pathways (Table 1, Table 2 and Table 3). These results may be expected, as our cells were subjected to only short-term siRNA treatment. But apoptosis and necrosis are closely related pathways, with shared contributors such as the mitochondria, ROS, and many protein factors [30,34,35,36], so the introduction of extrinsic signals or the use of other cell types such as immune cells might unveil the role of necrosis following CS loss and TCA cycle disruption.

In summary, our work reveals the important regulation of multiple cellular processes by CS and provides a framework for investigating CS’s contribution to the cell cycle, apoptosis, and mitochondrial functions and the underlying mechanisms in human cells. There are a number of limitations in this study that can be improved in the future. Firstly, carbohydrate metabolites were not measured, and cells were examined usually six days after siRNA transfection. While longer-term CS knockdown might be even more detrimental to cells, such an investigation is nevertheless worth pursuing. Secondly, due to the scope of this study, it is unclear whether the observed cellular phenotypes result directly or indirectly from reduced CS expression and if there are other alterations in cell behaviors. Thirdly, three cancer cell lines were examined here, but a comprehensive and systematic survey using cell lines from more cancer and cell types may provide a more definitive answer and/or elucidate cell-specific effects. Lastly, how results from cell line studies translate to in vivo situations is uncertain.

## 4. Materials and Methods

### 4.1. Plasmid Construction and siRNAs

The coding sequence of the human full-length CS (NM_004077.3) was synthesized and cloned into the p3xFLAG-CMV10 vector to produce pCMV-CS-3xFLAG (General Biology, Hefei, China). This plasmid expresses the CS protein tagged at its C-terminus by the 3xFLAG epitope. A control siRNA and a CS siRNA were ordered from and synthesized by Sangon (Shanghai, China). The CS siRNA (5′-GCGAGAGUUUGCUCUGGAAACA-3′) is identical to that used in [9].

### 4.2. Cell Cultures

The HCT116, HT-1080, and HepG2 cell lines were acquired from BD Bio (Hangzhou, China). HCT116 cells were cultured in McCoy 5A medium supplemented with 10% fetal bovine serum and 2 mM L-glutamine (Invitrogen, Waltham, MA, USA). HT-1080 and HepG2 cells were cultured in Eagle’s minimum essential medium supplemented with 10% fetal bovine serum and 2 mM L-glutamine. All cells were maintained at 37 °C with 5% CO_2_ in a cell culture incubator. Cells were transfected with a plasmid or siRNA, with approximately 400 ng of total nucleic acids per well in a 24-well plate, using Lipofectamine 2000 (Invitrogen).

### 4.3. Protein Analyses

Cell lysis, protein extraction, Western blotting, and image acquisition and quantification were performed as described [13]. The primary FLAG antibody was from Sigma (dilution 1:5000, catalog number F3165, St. Louis, MO, USA), GAPDH antibody from Solarbio (dilution 1:2000, catalog number K200057M, Beijing, China), and CS antibody from Solarbio (dilution 1:2000, catalog number K008664P).

### 4.4. Cell Phenotype Analyses

The MTT (catalog number A600799, Sangon) assay to measure cell proliferation has been described [18]. Approximately 2000–3000 cells were seeded per well in a 96-well plate and transfected with the control or CS siRNA, and cell numbers were measured once every 24–48 h. For time points longer than 3 days, cells were split 1:8 when confluent for further growth and observation.

The cell cycle profiles were examined using the Cell Cycle and Apoptosis Analysis Kit (YEASEN, Shanghai, China) [18]. Cells were transfected with siRNAs in a 24-well plate and, when confluent, transferred to a 6-well plate. Six days after transfection, cells were washed, trypsinized, and collected by centrifugation. Cells were fixed in cold 70% ethanol, washed, and stained with a PI solution containing RNase A. The stained cells were analyzed using a BD Accuri C6 flow cytometer and the FlowJo v10.6 software (Becton Dickinson, Franklin Lakes, NJ, USA). Cells at different phases of the cell cycle were determined automatically using the software’s default settings.

BrdU labeling and detection experiments were carried out to measure S-phase cells using the FITC-BrdU Cell Proliferation Detection Kit (KeyGen BioTech, Nanjing, China). Cells were transfected with siRNAs, and six days later, they were labeled with BrdU (Sigma) at 10 M for 60 min. Cells were then harvested, their DNA denatured by brief HCl treatment, washed, and incubated with a FITC-BrdU antibody. Cells were analyzed by flow cytometry as described above.

Apoptosis was assessed by using the Annexin V-Alexa Fluor 647/PI Apoptosis Detection Kit (YEASEN) [18]. Cells were transfected with siRNAs for 6 days. Cells were then harvested, stained with Annexin V-Alexa Fluor 647 and PI, and analyzed using a BD Accuri C6 flow cytometer and the FlowJo v10.6 software as described above.

The mitochondrial membrane potential was measured using the JC-1 Mitochondrial Membrane Potential Assay from Sciben (Nanjing, China) [18]. JC-1 is a dye that accumulates in the mitochondria. When the membrane potential is low, JC-1 exists as a monomer and produces green fluorescence. When the membrane potential is high, JC-1 aggregates yield red fluorescence. The ratio of red vs. green fluorescence can be an index of relative mitochondrial membrane potential changes. Six days after siRNA transfection, cells were stained with JC-1 solution, washed, and then analyzed using a BD Accuri C6 flow cytometer. Cells were excited at 490 nm and 525 nm and detected at 530 and 590 nm, respectively. The FlowJo v10.6 software was used to quantify red and green fluorescence signals.

ROS levels were measured using the Meilun Reactive Oxygen Species Assay Kit (Meilunbio, Dalian, China) [18]. The kit employs 2,7-Dichlorodi-hydrofluorescein diacetate, a dye that fluoresces when oxidized by ROS. Cells were transfected with siRNAs. Six days later, the cells were stained with the dye for flow cytometry studies, excited at 488 nm, and detected at 525 nm. Fluorescence signals were quantified using the FlowJo v10.6 software.

ATP levels were determined using the ATP Assay Kit from Beyotime (Shanghai, China). Six days after siRNA transfection, cells were lysed in the extraction buffer in the kit. A normalized amount of lysates/proteins was mixed with the ATP assay substrates, and signals were acquired using a luminometer.

All experiments had at least three biological replicates.

### 4.5. RNA-Seq and Data Analyses

Total RNA was isolated from transfected HCT116, HT-1080, and HepG2 cells using Trizol (Invitrogen). For RNA-seq (General Biology), 1 μg of total RNA was used to prepare a library. Poly(A) mRNA was enriched using oligo(dT) beads. mRNA fragmentation was performed using divalent cations at high temperature. Priming was performed with random primers. First-strand cDNA synthesis was carried out using reverse transcriptase, followed by second-strand cDNA synthesis. Double-stranded cDNA was purified, treated to repair ends, and then a dA-tail was added, followed by T-A ligation to introduce adaptors to both ends. Size selection of adaptor-ligated DNA was performed using DNA Clean Beads. Samples were then amplified by PCR. Finally, libraries were multiplexed and loaded on an Illumina HiSeq/Illumina Novaseq/MGI2000 instrument for sequencing using a 2 × 150 paired-end (PE) configuration according to the manufacturer’s instructions (Illumina, San Diego, CA, USA).

Raw sequencing reads in FASTQ format were treated with Fastp (v0.24.1) to remove technical sequences and low-quality bases. Sequences were processed by Cutadapt (V1.9.1), and the clean data were aligned to the human reference genome GRCh38 with HISAT2 (v2.2.1). Gene-level counts were quantified using HTSeq (v0.6.1). RNA-seq data have been deposited in the Gene Expression Omnibus under the accession number GSE306823. Student’s *t*-test was performed to identify differentially expressed genes (*p* < 0.05). Gene Set Enrichment Analysis (GSEA) [20] and DAVID (https://davidbioinformatics.nih.gov, accessed on 5 November 2025) [21] were used to analyze differences in biological processes and pathways.

### 4.6. RNA Isolation and Real-Time PCR (qPCR) Analyses

Total RNA was isolated from siRNA-transfected cells using Trizol. For mRNA expression analyses, the SuperScript III First-Strand Synthesis SuperMix (Invitrogen) was used to reverse transcribe RNA. qPCR reactions were then carried out on the QuantStudio 6 Flex Real-Time PCR System (Thermo Fisher Scientific, Waltham, MA, USA) using the SYBR Green qPCR Master Mix (Thermo Fisher Scientific). The relative expression of target genes was calculated with the 2^−ΔΔCT^ method, normalized to that of the Actin mRNA [18].qPCR primers were synthesized by Sangon:Actin: 5′-GGACTTCGAGCAAGAGATGG-3′ and 5′-AGCACTGTGTTGGCGTACAG-3′; IRF7: 5′-GCTGGACGTGACCATCATGTA-3′ and 5′-GGGCCGTATAGGAACGTG C-3′ [9]; ISG15: 5′-CGCAGATCACCCAGAAGATCG-3′ and 5′-TTCGTCGCATTTGTCCACCA-3′ [9]; DDX58: 5′-CTGGACCCTACCTACATCCTG-3′ and 5′-GGCATCCAAAAAGCCACGG-3′ [9]; CASP7: 5′-CGGAACAGACAAAGATGCCGAG-3′ and 5′-AGGCGGCATTTGTATGGTCCTC-3′; Casp3: 5′-GGAAGCGAATCAATGGACTCTGG-3′ and 5′-GCATCGACATCTGTACCAGACC-3′; Casp10: 5′-CCAGGCTATGTATCCTTTCGGC-3′ and 5′-TCGTTGACAGCAGTGAGGATGG-3′; BNIP3L: 5′-AATGTCGTCCCACCTAGTCG -3′ and 5′-CCCCCATTTTTCCCATTGCC-3′; PAWR: 5′-GGCAGAAAGAGCGGAAACGAGA-3′ and 5′-GCCTGAAACTGTTCTAGGTGGC-3′; CIAPIN1: 5′-GGCTCTGAAAGGTCTGGTGGAT-3′ and 5′-AGAGTGGTGCTTCCTGGGACTA-3′; AATF: 5′-GAGATGGAGGACTATCCCAGCT-3′ and 5′-GAGCGTTCAAAGGCACCAAAACC-3′; and PEA15: 5′-CCAGCGAAAAGAGTGAGGAGATC-3′ and 5′-TGTGCTCAATGTAGGAGAGGTTG-3′.

### 4.7. Statistical Analysis

GraphPad Prism 7.0 (GraphPad Software, San Diego, CA, USA) was used to perform the two-sided, two-sample unequal variance Student’s *t*-test and Dunnett’s test, with *p* value < 0.05 considered statistically significant.

## Figures and Tables

**Table 1:** Top 10 terms in the UP_KW_BIOLOGICAL_PROCESS category for the differentially expressed genes between the control and CS RNAi cells.

UP_KW_BIOLOGICAL_PROCESS	*p* Value
KW-0653~Protein transport	5.96 × 10^−5^
KW-0752~Steroid biosynthesis	9.09 × 10^−4^
KW-0945~Host-virus interactions	0.00251
KW-0498~Mitosis	0.00838
KW-0813~Transport	0.00954
KW-0443~Lipid metabolism	0.0100
KW-0810~Translation regulation	0.0115
KW-0159~Chromosome partition	0.0171
KW-0276~Fatty acid metabolism	0.0214
KW-0970~Cilium biogenesis	0.0234

**Table 2:** Top 10 terms in the GOTERM_BP_DIRECT category for the differentially expressed genes between the control and CS RNAi cells.

GOTERM_BP_DIRECT	*p* Value
GO:0015031~protein transport	4.05 × 10^−4^
GO:0007059~chromosome segregation	5.46 × 10^−4^
GO:0043066~negative regulation of apoptotic pathway	8.82 × 10^−4^
GO:0042149~cellular response to glucose starvation	9.25 × 10^−4^
GO:0007030~Golgi organization	0.00136
GO:0006094~gluconeogenesis	0.00224
GO:0045944~positive regulation of transcription by RNA polymerase II	0.00240
GO:0045893~positive regulation of DNA-templated transcription	0.00327
GO:0060271~cilium assembly	0.00449
GO:0006631~fatty acid metabolic process	0.00497

**Table 3:** Top 10 terms in the KEGG_PATHWAY category and BIOCARTA terms for the differentially expressed genes between the control and CS RNAi cells.

KEGG_PATHWAY	*p* Value
hsa04137:Mitophagy	4.94 × 10^−4^
hsa01100:Metabolic pathway	0.00333
hsa00100:Steroid biogenesis	0.00358
hsa00900:Terpenoid backbone biosynthesis	0.00680
hsa00592:alpha-Linolenic acid metabolism	0.0116
hsa03013:Nucleocytoplasmic transport	0.0136
hsa03083:Polycomb repressive complex	0.0137
hsa00591:Linoleic acid metabolism	0.0228
hsa04710:Circadian rhythm	0.0346
**BIOCARTA**	
h_caspasePathway:Caspase Cascade in Apoptosis	0.0176
h_tnfr1Pathway:TNFR1 signaling pathway	0.0431
h_hivnefPathway:HIV-I Nef: negative effector of Fas and TNF	0.0432

## Data Availability

RNA-seq data generated in this work have been deposited in the Gene Expression Omnibus under the accession number GSE306823.

## Supplementary Materials

The following supporting information can be downloaded at: https://www.mdpi.com/article/10.3390/ijms27010083/s1.
