# Supplementary material for: Citrate Synthase Knockdown Suppresses Cell Proliferation and Induces Apoptosis in Select Human Cancer Cell Lines
Source: Int J Mol Sci. 2025 Dec 21;27(1):83. doi: 10.3390/ijms27010083 (PMC12785315; doi:10.3390/ijms27010083)
Supplement: Supplementary file 1 [file ijms-27-00083-s001.zip › Suppl/CS suppl Figures.pptx]

## Slide 1
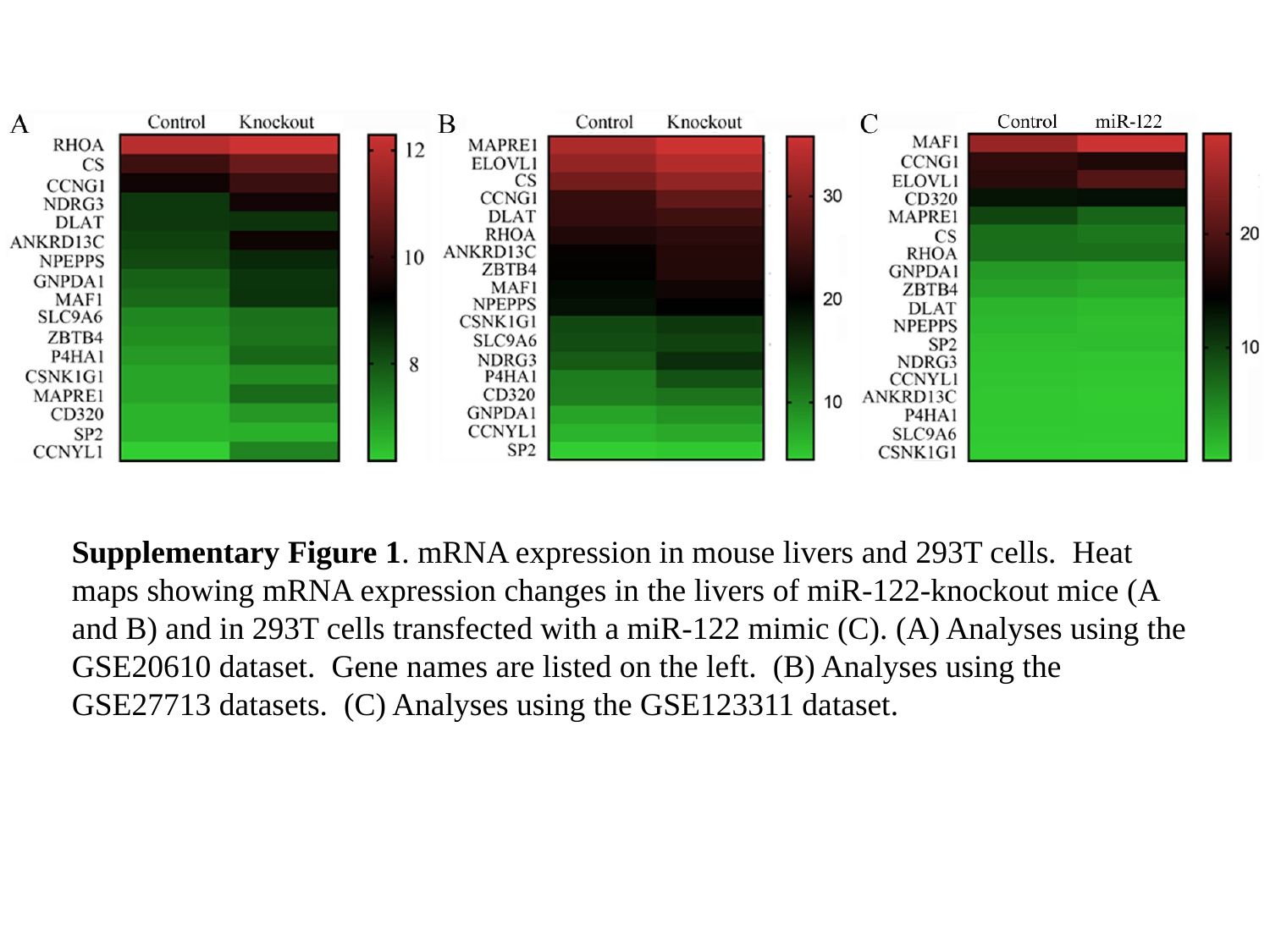

Supplementary Figure 1. mRNA expression in mouse livers and 293T cells. Heat maps showing mRNA expression changes in the livers of miR-122-knockout mice (A and B) and in 293T cells transfected with a miR-122 mimic (C). (A) Analyses using the GSE20610 dataset. Gene names are listed on the left. (B) Analyses using the GSE27713 datasets. (C) Analyses using the GSE123311 dataset.

## Slide 2
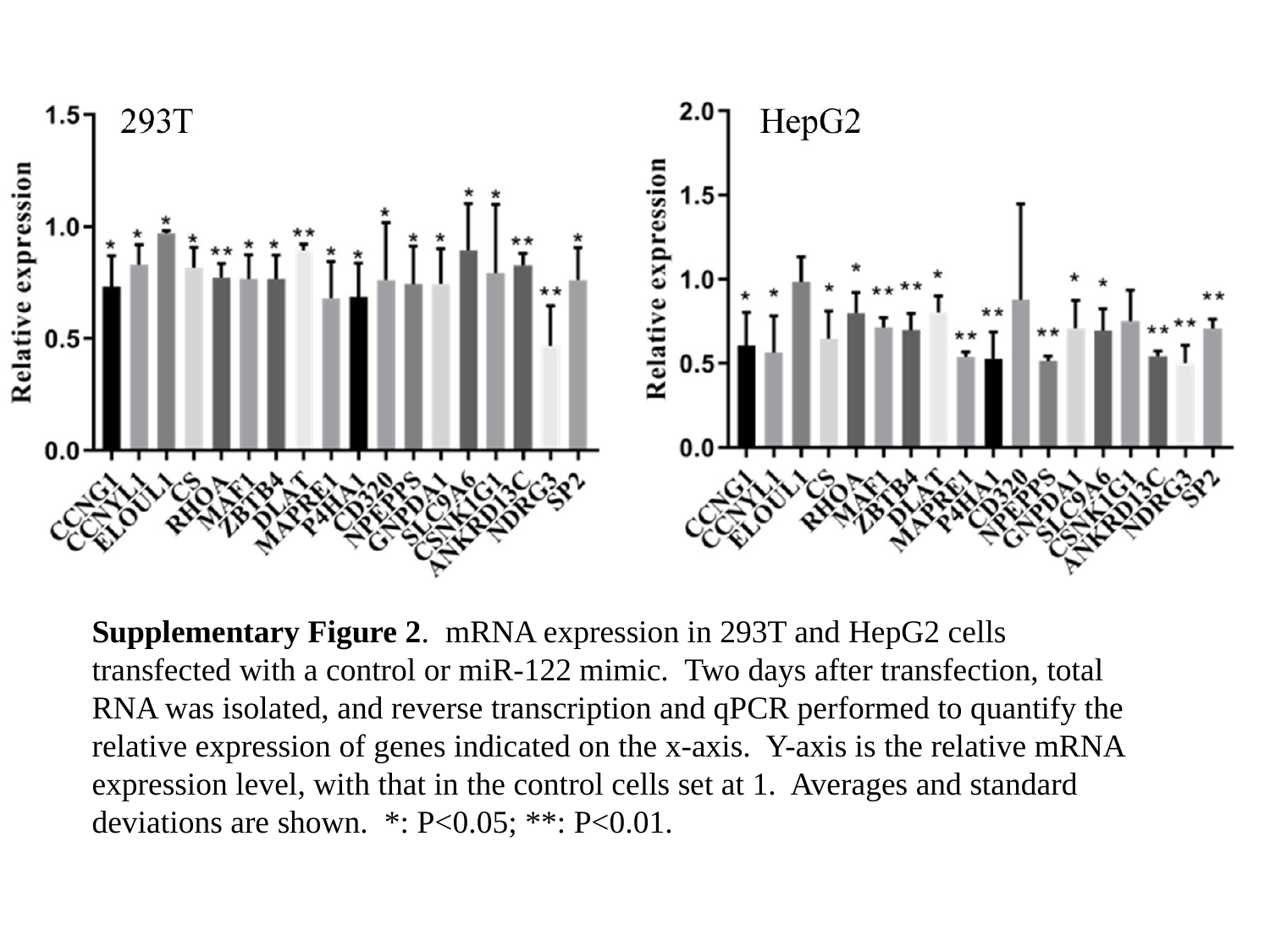

Supplementary Figure 2. mRNA expression in 293T and HepG2 cells transfected with a control or miR-122 mimic. Two days after transfection, total RNA was isolated, and reverse transcription and qPCR performed to quantify the relative expression of genes indicated on the x-axis. Y-axis is the relative mRNA expression level, with that in the control cells set at 1. Averages and standard deviations are shown. *: P<0.05; **: P<0.01.

## Slide 3
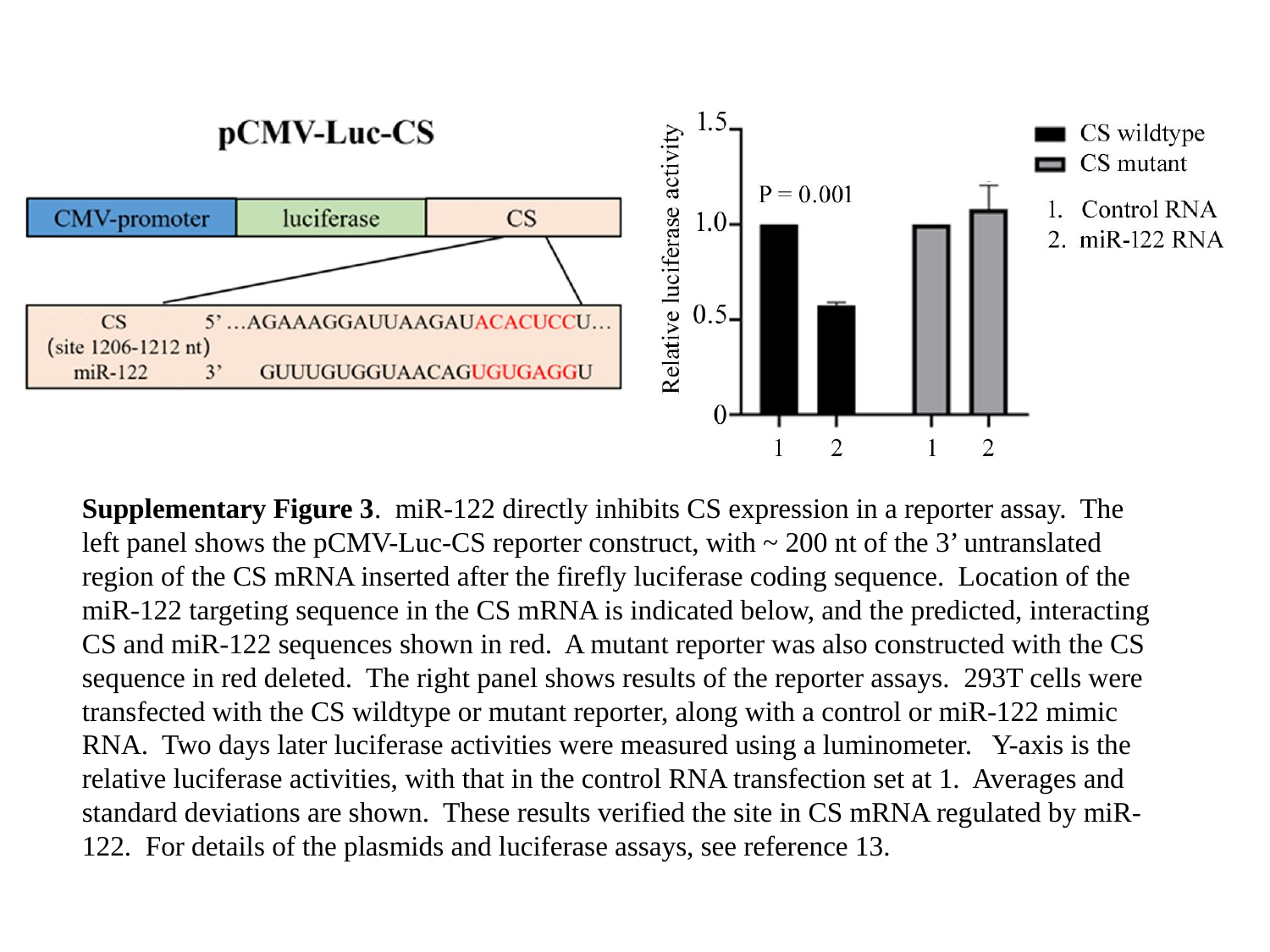

Supplementary Figure 3. miR-122 directly inhibits CS expression in a reporter assay. The left panel shows the pCMV-Luc-CS reporter construct, with ~ 200 nt of the 3’ untranslated region of the CS mRNA inserted after the firefly luciferase coding sequence. Location of the miR-122 targeting sequence in the CS mRNA is indicated below, and the predicted, interacting CS and miR-122 sequences shown in red. A mutant reporter was also constructed with the CS sequence in red deleted. The right panel shows results of the reporter assays. 293T cells were transfected with the CS wildtype or mutant reporter, along with a control or miR-122 mimic RNA. Two days later luciferase activities were measured using a luminometer. Y-axis is the relative luciferase activities, with that in the control RNA transfection set at 1. Averages and standard deviations are shown. These results verified the site in CS mRNA regulated by miR-122. For details of the plasmids and luciferase assays, see reference 13.

## Slide 4
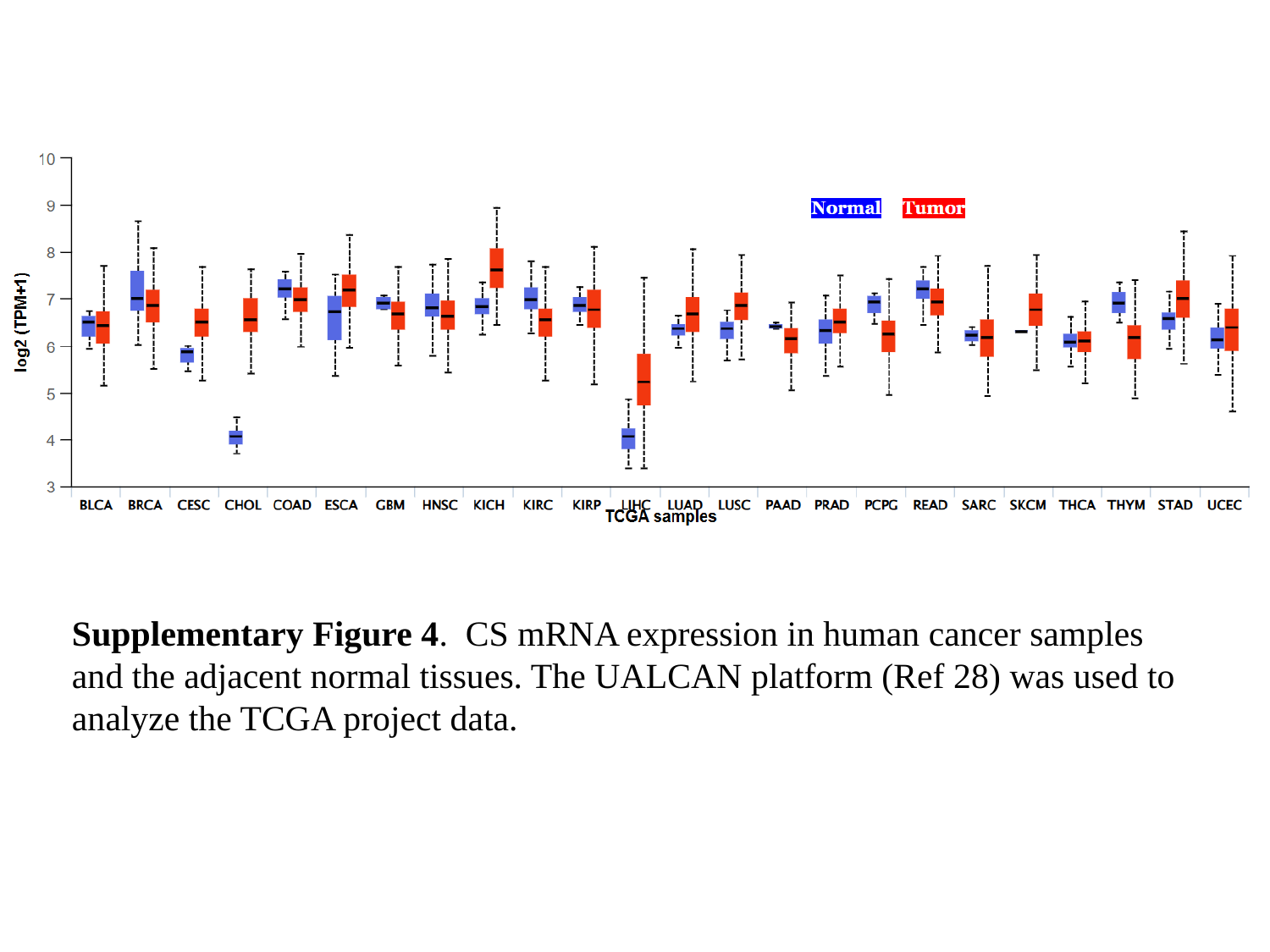

Supplementary Figure 4. CS mRNA expression in human cancer samples and the adjacent normal tissues. The UALCAN platform (Ref 28) was used to analyze the TCGA project data.
